# Stem Cell Tracking with Nanoparticles for Regenerative Medicine Purposes: An Overview

**DOI:** 10.1155/2016/7920358

**Published:** 2015-12-29

**Authors:** Lisa Accomasso, Clara Gallina, Valentina Turinetto, Claudia Giachino

**Affiliations:** Department of Clinical and Biological Sciences, University of Turin, Regione Gonzole 10, Orbassano, 10043 Turin, Italy

## Abstract

Accurate and noninvasive stem cell tracking is one of the most important needs in regenerative medicine to determine both stem cell destinations and final differentiation fates, thus allowing a more detailed picture of the mechanisms involved in these therapies. Given the great importance and advances in the field of nanotechnology for stem cell imaging, currently, several nanoparticles have become standardized products and have been undergoing fast commercialization. This review has been intended to summarize the current use of different engineered nanoparticles in stem cell tracking for regenerative medicine purposes, in particular by detailing their main features and exploring their biosafety aspects, the first step for clinical application. Moreover, this review has summarized the advantages and applications of stem cell tracking with nanoparticles in experimental and preclinical studies and investigated present limitations for their employment in the clinical setting.

## 1. Introduction

The main purpose of regenerative medicine is to restore damaged or ageing tissues by mimicking their native morphology and function. In this concern, during the last years, advances in this field have been strictly correlated with new and promising approaches in tissue engineering, bioengineering, nanotechnology, and stem cell (SC) biology, thereby addressing extremely topical issues from a marked interdisciplinary perspective [1].

The newest therapeutic strategies in regenerative medicine are often directed to favor the intrinsic self-regenerating ability of the tissues and thus principally rely on techniques based on the use of specific soluble growth factors, biomaterials, and especially stem or progenitor cells (SCs/PCs).

Indeed, to ensure that these treatments are a success, it is essential to determine the fate of SCs, their functional capabilities, and the biological role that they play.

In this review, we will first introduce the most relevant cell types for regenerative medicine purposes; then, we will elucidate the main features of the available nanoparticles (NPs) for SC tracking, focusing on their biosafety aspects; lastly, we will describe some examples of NP applications for fluorescent, magnetic resonance and photoacoustic imaging of SCs in* in vivo *models.

## 2. Stem Cells for Regenerative Medicine Purposes

SCs can be defined as unspecialized cells capable of both self-renewal potential, that is, the ability to retain their stemness through controlled proliferation, and commitment to differentiation into one or more mature cell types in the body [2].

For the purpose of regenerative medicine approaches, SCs should answer to specific criteria: (a) great availability, that is, SCs should be found in abundant quantities; (b) noninvasive procedures to harvest them; (c) regulated and reproducible ways to gain specific differentiated cell lineages from starting SCs; (d) efficient and safe autologous or allogeneic transplantations into patients; and (e) manipulation in accordance with the “Good Manufacturing Practice” guidelines [3].

Among the several suitable SCs populations,* embryonic stem cells* (ESCs) were first isolated from mouse embryos [4, 5] and can be defined as a pluripotent cell lineage deriving from the epiblast tissue of the inner cell mass of the blastocyst. Although this population has been extensively used in regenerative medicine, several studies underlined ethical problems for its clinical application [6, 7]. Other works then proposed the use of the more upstanding* induced pluripotent stem cells* (iPSCs), that is, somatic cells that are reprogrammed for pluripotency via the overexpression of a specific set of genes [8–11]. Nevertheless, the main issue for both ESCs and iPSCs is the ability to form teratomas [12–14], which are considered a major obstacle for biomedical applications [15]; in addition, iPSCs have also been associated to marked tumorigenic activity [16].

Besides pluripotent SCs, in the adults, many organs posses tissue-specific populations of SCs which can give rise to differentiated cell lineages appropriate for their location, therefore not fulfilling the principle of pluripotency and, with respect to ESCs and iPSCs, being less self-renovating [17]. Among the different tissue-specific SCs, including hematopoietic [18] and neuronal [19] SCs,* mesenchymal stem cells* (MSCs) are probably the most important population applicable in human regenerative medicine.

MSCs are defined as a population of multipotent stromal cells that can be isolated from a variety of both adult and fetal tissues, including bone marrow [20], still the major source, adipose tissue [21], placenta [22], and umbilical cord [23], with the capability to differentiate, under appropriate conditions, into chondrocytes, osteoblasts, and adipocytes and to commit to neurons, cardiomyocytes, and endothelial cells [17, 20, 24–27].

Unlike ESCs and iPSCs, MSCs do not have ethical problems, can be easily obtained in large amounts from patient's own tissue (especially bone marrow and fat), and present an extremely low risk of tumorigenesis, although they are not completely free of malignant transformation [28]. MSCs have been proposed as a powerful tool for the treatment of various pathologies, including immune and degenerative disorders [29, 30], and prevention of left ventricular remodeling after myocardial infarction [31].

During the past years, it was believed that the therapeutic outcome of transplanted MSCs was principally due to cell engraftment and differentiation at the site of injury. However, only a small percentage of delivered MSCs survive and engraft after transplantation, while it has become evident that these cells exert positive effects on the host tissue by preferentially secreting a variety of paracrine/autocrine factors, the so-called secretome [32], which may generate in the injured tissue a microenvironment that can support regenerative processes, induce angiogenesis, and protect against further tissue death [29, 33]. Additionally, transplanted MSCs have demonstrated immunomodulatory properties, low immunogenicity, and the capability to reduce oxidative stress and inflammation by direct interaction with neutrophils, macrophages, and monocytes [31].

In addition to direct transplantation or injection, MSCs may also be delivered through the implant of biocompatible natural or synthetic scaffolds made up to mimic the regulatory characteristics of natural extracellular matrices (ECMs) and ECM-bound growth factors [34]. There are generally three main methods to generate MSCs/scaffold grafts: (a) cells can be expanded* in vitro* and seeded on the scaffold before implantation into the body's patient [35–38], (b) several days before transplantation, undifferentiated MSCs can be loaded onto the scaffold and induced to differentiate towards a specific lineage [39–41], and (c) the scaffold can be functionalized with SC-attractive molecules and then acellularized scaffolds are implanted allowing* in situ* regeneration through recruitment of autologous cells [42, 43].

Advances in preclinical research demonstrating the safety and feasibility of MSCs transplantation in different pathologies, such as myocardial infarction, stroke, regeneration of bone and cartilage defects, spinal cord injury, graft-*versus*-host disease, and blood disorders, have paved the way to translation of MSC-based therapies in clinical settings all over the world [44]. Although several clinical trials employing MSCs transplantation have been listed in public databases, among them ClinicalTrials.gov for USA (http://clinicaltrials.gov/) and Clinical Trials Register for EU (https://www.clinicaltrialsregister.eu/index.html), however, still few approaches have been translated into humans. One reason is that, in order to assess the outcome of therapies with SCs and to develop more efficient therapies, the clinicians need to determine in real time the physiological state of engrafted cells and to monitor their survival in patients. However, the exact mechanisms at the base of SC distribution and engraftment and, especially, the balance within these processes remain elusive [45, 46]. Indeed, the often poor survival of engrafted cells, due to an adverse host tissue microenvironment or to inadequate nutrient support, may compromise the outcome of SC therapies [47].

Based on these considerations, during the last few years, several research groups examined safe ways to track the movement of the implanted SCs in the target tissue and generally inside the host with the attempt to guarantee long-term analysis of cell survival, migration, redistribution, and differentiation, besides understanding the best injection method for cell delivery.

## 3. A Brief Overview on Stem Cell Tracking Methods

Traditional methods to follow implanted SC fate forecasted* in vitro* cell labeling before cell transplantation and subsequent follow-up of cell engraftment and survival through the analysis of histological sections coming from sacrificed animals or tissue biopsies, an invasive technique that did not permit long-term and continuous analyses [48, 49].

Recent advances in SC therapy require more accurate and noninvasive methods for qualitatively and quantitatively monitoring transplanted cells inside the host, in order to facilitate the understanding of treatment outcomes and ultimately improve patients handling [50].

The most common techniques take advantage of specific contrast agents, such as endogenous biomolecules with intrinsic fluorescence [51], exogenous fluorescent proteins [52], or nonfluorescent organic dyes [53], which have been used for essentially two labeling modalities: (a) direct labeling, that is, cell incubation with specific intracellular probes, and (b) indirect labeling, through the expression of the indicator by a reporter gene inserted in the genome of the cells. Direct methods are easy to apply and less expensive, however, potential limitations include fast signal decay due to cell proliferation and subsequent insufficient marker distribution between daughter cells. On the other hand, indirect techniques are much more stable but require genetic manipulation of cells and may not be suitable for clinical applications [54].

In general, the majority of the employed contrast agents often present disadvantages like photo-bleaching over time, interference derived from tissue autofluorescence, chemical and/or metabolic degradation* in vivo, *and even low transfection efficiency in primary cells and thus are not considered suitable for* in vivo* imaging. To overcome this limitation, several engineered NPs with unique magnetic and/or optical properties have been developed and employed in biomedicine, due to their capability to offer real-time methods of tracking intracellular processes at a biomolecular level [55–57].

## 4. Nanoparticles to Track Stem Cells

Over the last years, the definition of “nanoparticle” or “nanomaterial” has been controversial. Currently, the most used criterion to define NPs is size [58, 59]; in October 2011, the European Commission (EC) published a recommendation (2011/696/EU) [60] on the definition of “nanomaterial” as “a natural, incidental or manufactured material containing particles, in an unbound state or as an aggregate or as an agglomerate and where, for 50% or more of the particles in the number size distribution, one or more external dimensions is in the size range 1 nm–100 nm”. However, other parameters have been used; for example, a more recent definition focused on the importance of NP surface area; indeed, as the particle size decreases, its specific surface area increases allowing a greater proportion of its atoms to be displayed on the surface. Focusing on this characteristic, the new definition indicates that a NP should have a surface area > 60 m^2^/cm^3^ [61, 62]. New synthesis techniques allowed for producing not only spherical NPs, but also other shapes such as cube [63, 64], prism [65, 66], hexagon [64, 67], octahedron [68], rod [69, 70], and tube [71]. Importantly, morphology and size determine the physicochemical properties of the NPs, as they lead to different cellular uptake and interaction with biological tissues that would not be possible using the bulk material [72].

Before practical use, NPs should undergo a comprehensive characterization in terms of purity, that is, the fraction of NP composition beyond chemical and biological contaminants, and physicochemical features in both dry and wet conditions [73, 74]. Furthermore, it is commonly accepted that the contact of NPs with a biological environment leads to the adsorption of biomolecules such as proteins and lipids on the material surface. The possibility that this interface's events implicate a constitution of new bioactive sites on NPs surface has been the focus of several papers [75, 76]. Interestingly, different studies have highlighted that this biological* corona* exposes specific epitopes that are specific to the NP surface propriety [77, 78] and to the time and the biological media in which they are exposed [79–82].

Besides tracking living transplanted cells [83], which represents the target of the present review, engineered nanomaterials have also being exploited for other applications, among them, industrial products such as topical sunscreens and cosmetics, inks food products, and toothpastes. In the context of biomedical application, NPs have been investigated for drug or gene delivery [84–86] and for nanotheranostics [87], a branch of medicine that can be applied to noninvasively discover and target markers and deliver treatments based on biomarker distribution, in order to gain both diagnosis and therapy for several pathologies [88]. In particular, some NPs are currently used in clinical trials for cancer thermal therapy [89–91]. Despite the great interest in these promising applications, the focus of the present review is to introduce to the readers the application of NPs for SC tracking.

## 5. Biosafety Profiles of Nanoparticles Used to Track Stem Cells

In general, NP technology applied for* in vivo* noninvasive SC tracking must allow long-term and sensitive localization of the cells avoiding cytotoxicity as much as possible. However, it is important to underline that almost no NPs have been used for therapeutic SC tracking into patients yet. The reason is that, before any approval for clinical use, there must be a fundamental step of NP characterization for both chemical composition and biological effects on SCs, including viability rate after loading, influence on SC migration, differentiation, engraftment, and evaluation of possible short- and long-term cytotoxicity.

Nanotoxicology is the branch of toxicology born to precisely address the adverse effects caused by nanomaterials, in order to contribute to the development of a sustainable and safe nanotechnology [92]. Indeed, this discipline studies NP-induced toxicity in* in vitro* as well as* in vivo* experimental models and attempts at optimizing well known toxicity tests or producing new ones to be applicable for nanosafety evaluation [73, 93].

In general, toxic effects on cells induced by NP uptake may depend on particular characteristics of the NP itself; for example, with respect to the bulk material, NPs possess higher surface area to volume ratio and surface reactivity and are more susceptible to degradation or ion leaching [94, 95]. Moreover, frequent NP agglomeration and/or sedimentation might influence subsequent uptake and lead to cytotoxicity [96].

The first mechanism of NP-induced cytotoxicity may derive from how NPs enter the cells. Indeed, some NPs can be internalized via passive diffusion and possibly lead to toxicity by directly interacting with the cell cytosol, its structures, and/or DNA; most types of NPs are instead endocytosed by cells and confined through clustering in cytoplasmic vesicles, especially lysosomes or late endosomes [97, 98]. However, some NPs might be susceptible to the oxidative environment of these organelles and thus undergo degradation or dissolution, resulting in the leaching of free ions or an increase in reactive surface groups.

A second potential NP toxicity mechanism is actin cytoskeleton disruption. Due to endocytosis events, the cells undergo a reorganization of cytoskeleton [99] which plays a major role in fundamental cellular physiology aspects of the cell such as shape, motility, division, adhesion, and connection with the surrounding environment [100]. The relation between endocytosis and cytoskeleton organization is highly dynamic and may involve interactions between distinct protein complexes [101]. As a result, different pathways of NPs internalization might lead to the modification of proteins normally responsible for maintaining cytoskeleton organization [102]. Furthermore, the aberration in cell morphology could result in a decrease in cell migration [103]. It has to be underlined that the disruption of actin cytoskeleton is often coupled [104] with the main molecular mechanism of NP-induced toxicity, that is, the enhancement of oxidative stress via incremented production of reactive oxygen species (ROS) [105]. ROS may be generated directly from free radicals on the surface of NPs; otherwise, NPs constituted of transition metals like iron NPs may generate ROS (in particular, hydroxyl radicals) by acting as catalysts in Fenton-type reactions [106].

Other toxic effects caused by NP uptake, which seem to be secondary to altered ROS production, are (a) alterations of gene expression mediated by either direct NP-induced DNA damage [94, 107, 108] or interaction of NPs with the cellular transcription/translation machinery after perinuclear localization [108, 109], (b) morphological modifications such as protein denaturation, modulation of intracellular signaling cascades, or membrane damage [94], and (c) immunological effects through the upregulation of redox-sensitive transcription factors or proinflammatory kinases [110] or the initiation of an immune response directed to specific proteins localized in the outside NP* corona* [111].

Over the last years, research groups that study the outcomes of NPs used for SCs tracking are focusing on their possible undesirable effects inside the experimental model or even the host. Thus, prior to the therapeutic use of NPs, it is becoming increasingly important to conduct systematic* in vitro* studies to assess their toxicological profiles and evaluate their potential influence on the self-renewal and differentiation properties of SCs [112, 113].

To show in further detail some examples of biosafety study of specific nanomaterials in contact with SCs, next part of this review will focus on selected NPs representing the most studied products for cell tracking: quantum dots (QDs), silica NPs, and polymer NPs that are the most used for fluorescence imaging due to their advantages regarding quantum yield, brightness, and photostability; superparamagnetic iron oxide (SPIO) NPs, which are the most significant for magnetic resonance imaging; and gold (Au) NPs that are useful for photoacoustic imaging. In Table 1, we summarize these different studies.

### 5.1. Quantum Dots

QDs are highly fluorescent semiconductor NPs, composed of materials from the elements in the periodic groups of II_VI, III_V, or IV_VI, such as indium phosphamide (In from group III and P from group V) and cadmium telluride (Cd from group II and Te from group VI), and they usually have diameter around 2–10 nm [114]. QDs possess unique optical and physical properties like narrow and tunable emission spectra, exceptional photochemical stability, high quantum yields, and multiplex imaging due to simultaneous excitation [115, 116]. These platforms have been incorporated into various assays for the reversible detection and quantification of biomolecules [117–119] in applications such as immunoassays [120, 121] and molecular diagnosis [122] and in clinical assays [123, 124]. When applying QDs to biological imaging and cellular studies, the toxic nature of Cd-containing QDs remains a major concern [125]. For the preparation of biocompatible QDs, the coating of the CdSe core, with, for instance, a ZnS layer, is indispensable [126–128] and the quality of the coating might determine the toxic effects on cultured cells. However, thanks to the recent advances in the development of surface modifications and to the production of Cd-free QDs [126–128], the potential toxicity of Cd itself is long a problem for* in vitro* and* in vivo* imaging studies with QDs.

Based on their favorable properties, QDs have been used since 1998 [129, 130] for bioimaging applications, in particular, to label different cell lines for both* in vitro* and* in vivo* studies. Common methods used for an efficient intracellular delivery of QDs are microinjection, electroporation, lipid-based transduction, and peptide mediated delivery.

The effects of QDs on SC self-renewal and differentiation are largely unknown, particularly in embryonic SCs. Some studies have reported no adverse effects on SC morphology, viability, proliferation, or differentiation over the duration of the experiments (from hours to several days) at QD concentrations optimized for labeling efficiency [131, 132], while others have noticed alterations in the differentiation profile of SCs [133, 134] and abnormalities during embryo development [135]. Therefore, QDs are not completely innocuous, but there is likely to be a safe range within which they can accomplish their task without major interference in the processes under study [135].

Perhaps the broadest effort to study the cytotoxicity of QDs in SCs has been made using human-derived MSCs (hMSCs). Some studies have reported that QDs do not affect cellular proliferation or cell-cycle distribution but do affect chondrogenic and osteogenic differentiation potential [133, 134]. However, recent findings indicate that QDs do not interfere with the differentiation program of SCs [131, 132]. hMSCs labeled with a range of external doses of QDs conjugated with a cell-penetrating peptide (cholera toxin) from 250 pM to 16 nM maintained their osteogenic differentiation potential. The cells showed upregulation of alkaline phosphatase activity, an early osteogenic marker, when cultured in osteogenic media and expressed the osteogenic gene* Osterix* after exposure to BMP-2 [131]. In another study [132], hMSCs labeled with a concentration of 20–50 nM QDs were viable and continued to proliferate for at least 22 days while retaining QDs in their cytoplasm. Furthermore, no interference of QDs was detected in the differentiation of hMSCs into osteogenic, chondrogenic, and adipogenic cell lineages [132]. More recently, Wang et al. [136] labeled MSCs isolated from the human amniotic membrane with different doses of QDs and investigated their effects from 1 to 4 days, observing that the concentration of 0.75 *μ*g/mL did not produce morphological modifications and alteration in expression of specific surface antigens such as CD29, CD44, CD90, and CD105, while cells maintained a viability > 80% [136].

An advantage of cell labeling with QDs is that they can concurrently tag multiple inter- and intracellular components for time ranging from seconds to months and, by using different coloured QDs, several cell components can be visualized with fluorescent microscopy or* in vivo* [137]. In this concern, Shah and Mao [137] provided a detailed protocol to label selected integrins on the cell membrane of hMSCs with bioconjugated QDs by optimizing precise concentration and incubation time. Interestingly, authors discovered that bioconjugated QDs effectively labeled hMSCs not only during population doublings, but also during multilineage differentiation into osteoblasts, chondrocytes, and adipocytes. In addition, undifferentiated and differentiated SCs labeled with bioconjugated QDs could be readily imaged by fluorescent microscopy.

In a recent study, Chen et al. [138] used silver sulfide (Ag_2_S) QDs-based second near-infrared window (NIR-II 1.0–1.4 *μ*m) imaging to label hMSCs seeded on three-dimensional (3D) collagen scaffolds. They first assessed cell proliferation using 3-(4,5-dimethylthiazol-2-yl)-2,5-diphenyltetrazolium bromide (MTT) assay and observed negligible difference of viability between control and labeled hMSCs during 30 days of culture on the scaffolds. Furthermore, the pluripotency-associated transcription factors Nanog, Rex-1, Oct4, and Sox2 were expressed at similar levels with respect to unlabeled cells and finally both osteogenic and adipogenic differentiation of 3D-cultured hMSCs labeled with Ag_2_S QDs were not different form the control counterparts. After demonstrating that the multipotentiality of Ag_2_S QDs-labeled hMSCs grown on 3D collagen scaffolds was maintained, they have applied their labeled cells in an* in vivo* experiment that will be elucidated in the next section of this review.

### 5.2. Silica NPs

Silica NPs are widely applied in chemical industry, agriculture, and cosmetics. In addition, they are being developed in medical uses including diagnosis and therapy [139, 140], controlled release drug delivery, and gene transfection [141]. Such medical approaches have numerous applications including skin cancer therapy [142–144], transdermal drug delivery [145, 146], and gene delivery through transcutaneous vaccination [147]. They can act as carriers for drugs with low solubility and might improve drug safety, stability, and performance [148].

For SCs tracking purposes, silica NPs usually contain organic dye molecules for fluorescence imaging. In this system, silica acts as a matrix to chemically and physically confine the fluorescent dyes. Indeed, the silica partially protects the dye molecules from external quenchers, enhances the photostability of incorporated dyes, and, in some cases, provides a biocompatible and easy-to-functionalize surface for bioconjugation [149]. Fluorescent silica NPs are mainly made through two approaches: sol-gel or reverse microemulsion. Sol-gel method usually generates fluorescent silica NPs in the hundreds of nanometers to micron range [150], while reverse microemulsion strategy provides NPs with diameters from nanosize to micro size [151–153]. In both methods, the dye molecules encapsulated inside silica NPs determine their spectral characteristics.

Cyanine dye-doped silica NPs were reported to label hMSC without affecting viability, proliferation, stemness surface marker expression, and differentiation capability into osteocytes [154]. Moreover, the used silica NPs were able to directly discriminate between live and early-stage apoptotic SCs (both mesenchymal and embryonic) through a distinct external cell surface distribution which makes them ideal for SC labeling and tracking [154].

A study performed on different cell types [155] demonstrated that the presence of serum in the cell medium induced the formation of* corona* on silica NPs, with subsequent influence on their cellular uptake level. To evaluate if the presence and doses of serum during incubation could influence the uptake level in hMSC, Catalano et al. [156] recently elucidated the factors ruling the uptake of dye-doped silica NPs by hMSCs, suggesting different uptake mechanisms involved.

Although the main application of mesoporous silica NPs is drug delivery [141], there are few studies which used them for SC tracking. Huang et al. [157] observed the internalization of mesoporous silica NPs conjugated with fluorescein isothiocyanate by hMSCs, demonstrating that there were no differences in cell viability, proliferation, and immunophenotypic profiles of surface markers with respect to unlabeled cells. Furthermore, cells retained their capability to differentiate into adipocytes, chondrocytes, and osteocytes after NPs internalization. In addition, the uptake mechanism of these NPs by hMSCs was analysed via different inhibitors and the roles of clathrin-mediated endocytosis and actin-dependent endocytosis were demonstrated to be prevalent [157, 158]. The same group in 2008 [159] reported that silica NPs can enhance actin polymerization in MSCs and that the uptake of mesoporous silica NPs into hMSCs did not affect their osteogenic differentiation.

So far, there are no reports applying fluorescent silica NPs for* in vivo* SC tracking. However, on the basis of the biocompatibility observed especially with MSCs, during the last years, silica has been employed to cover different types of NPs and enhance their biocompatibility and cellular uptake, maintaining the core material characteristics for detection [160]. For example, in 2008, Liu et al. [161] demonstrated that silica-coated core-shell superparamagnetic iron oxide (SPIO@SiO_2_) NPs cocondensed with FITC-incorporated mesoporous silica were able to enhance the uptake efficiency of hMSCs, without affecting their viability and differentiation potential. Similarly, Zhang et al. [162] demonstrated that mesoporous silica coating facilitated cell uptake of SPIO NPs and improved cell labeling efficiency in a neuronal progenitor cell line, with no adverse effects on cell proliferation under labeling conditions.

Silica NPs have been shown to be useful as ultrasound contrast agents [163] and even to enhance the photoacoustic signal generated by gold NPs [164]. Recently, different gold NPs have been produced with a silica layer on the gold core, in order to obtain silica coating, and their characteristics and biosafety will be further detailed in the “gold NPs” section.

### 5.3. Polymer NPs

Polymer NPs are generally prepared through either dispersion of preformed polymers or polymerization of monomers [165]. Dendrimers, microgels, and modified polysaccharide NPs are new polymeric particles having several medical and practical applications, mostly used as anticancer drugs, drug and gene delivery carriers, and magnetic resonance imaging contrast agents once complexed with Gd(III) [166–170].

For SC tracking in regenerative medicine, fluorescent organic dyes are commonly used and they could be either physically entrapped in the polymer interior during the preparation of NPs or covalently bound to the polymer chain before the preparation of NPs. Currently, the most common fluorescent polymer NPs are polystyrene (PS) NPs that are mainly prepared through the emulsion polymerization method [171]. The uptake of polystyrene NPs by MSCs was investigated by Jiang et al. [172] using confocal fluorescence microscopy and flow cytometry. Two types of aminated particles, PS and NPS (100 nm in diameter), were synthesized by miniemulsion polymerization process. Both were functionalized with amino groups by addition of cetyltrimethylammonium chloride (CTMA) as surfactant, but a further covalent amino functionalization was carried out only for NPS by addition of 2-aminoethyl methacrylate hydrochloride (AEMH). The presence of two different types of amino groups on NPS surface (physically and covalently bound by addition of CTMA and AEMH, resp.) did not result in a significant change of *ζ*-potential values with respect to PS particles but induced a different behavior of those particles in relation to the internalization pathway. To assess the relative importance of specific endocytosis mechanisms, uptake was observed in the presence of the inhibitor drugs dynasore and chlorpromazine. Authors found that NPSs were rapidly internalized and accumulated to a much higher level in MSCs than PS NPs, predominantly via clathrin-mediated pathway, whereas the latter were internalized mainly via clathrin-independent endocytosis. The pronounced difference in the internalization behaviour of PS and NPS platforms points to specific interactions of the amino groups on the NP surface with the endocytosis machinery of the cells [172].

In addition to PS, fluorescent polymer NPs were also prepared with conjugated fluorescent polymers exhibiting amplified fluorescence responses, such as poly(arylenediethynlenes) [173], poly(3,4-ethylenedioxythiophene) [174], poly(thiophene-3-yl-acetic acid) [175], and polyacetylene [176].

However, despite their wide applications, PS NPs suffer from low incorporation and inadequate protection of the dye molecules, with consequent leaching, quenching, and photobleaching of the fluorophores [177], all main disadvantages for their application in long-term SC tracking.

Recently, a general strategy was reported to enhance the photostability of organic fluorophores for bioimaging applications and, as a proof of concept, bright and robust fluorescence was observed in solid states of a well-defined synthetic polymer polycaprolactone (PCL) consisting of di-(thiophene-2-yl)-diketopyrrolopyrrole (DPP) covalently linked in the middle of the PCL chain as a biocompatible and bioresorbable matrix [178]. PCL-DPP-PCL NPs were prepared through a nanoprecipitation process of these polymers and could be internalized by both tumor cells and SCs with little cytotoxicity.

Reports using fluorescent polymer NPs for SCs labeling and tracking are still few, but inorganic NPs grafted with polymer are an area of special interest because of the enhanced properties of both polymers and NPs. In their review, Francis et al. [179] covered the general topics of polymer grafted NPs and their preparation, properties, and applications.

### 5.4. Superparamagnetic Iron Oxide NPs

SPIO NPs are composed of an iron oxide core, generally of magnetite Fe_3_O_4_ or maghemite *γ*-Fe_2_O_3_, a coating layer and surface functional groups that provide NPs with hydrophilicity and stability and prevent NP aggregation [180, 181]. A coating layer of different materials, among them, dextran, chitosan, and gelatin, has been used to stabilize the internal magnetic core [182]. SPIO NPs act as good contrast agents in MRI, enhancing the contrast between different tissues present by inducing a darker area (negative contrast). One important feature of SPIOs is that they lose their magnetization vector induced by a magnetic field applied and become highly dispersed when the magnetic field is switched off [183].

SPIO NPs are composed of iron that might be reused/recycled by cells via the principal biochemical pathways deputed for iron metabolism. Potential mechanisms of iron-mediated toxicity thus include generation of iron-catalyzed ROS [184, 185].

So far, SPIOs are the only commercial NPs that have been regulated for clinical applications [186]. Among commercially available SPIO NPs, the most used as contrast agents for SC tracking were ferumoxides (Endorem in Europe and Feridex in the USA, coated with dextrans, with a particle size of 120 to 180 nm) and ferucarbotran (Resovist, coated with carboxydextrans, hydrodynamic diameter 62 nm), due to their approval by the Food and Drug Administration (FDA) in 2009 for clinical use as liver-specific contrast agents. Unfortunately, the manufacturers ceased commercial production of both agents due to commercial reasons. The clinical development of Ferumoxtran-10 (Combidex in the USA, Sinerem in Europe), designed for lymph node metastasis evaluation, is currently stopped [187]. NC100150 (Clariscan) is made of SPIO crystals of a magnetite/maghemite with low molecular weight polyethylene glycol coating on a carbohydrate residue and its development was discontinued because of safety issues. Currently, only one type of SPIOs (ferumoxytol, Feraheme) is marketed for the treatment of iron deficiency anemia in adult patients with chronic kidney diseases, rather than for imaging.

Usually, to facilitate incorporation into the cell, SPIOs are cross-linked with a membrane-translocating signal peptide (e.g., HIV-1 Tat protein) [188] or incubated in combination with transfection agents [189].

The cytotoxicity of SPIOs has been evaluated in different types of SCs, including ESCs [190–192], hMSCs [193, 194], and neural SCs (NSCs) [195–197]. In most cases, internalization of these NPs did not affect cell viability and growth. The survival rate of SCs cultured in a medium containing SPIOs was very high (97%–99%) indicating that these NPs did not affect cell viability [198–201]. So far, only one study has demonstrated that the internalization of SPIOs impaired the differentiation of SCs. Bulte et al. [202] reported that SPIOs (ferumoxides) uptake by hMSCs (intracellular iron incorporation of 13–16 pg Fe/cell), in the presence of the transfection agent poly-L-lysine, impaired their chondrogenic differentiation. Although the results of this study suggest that the inhibition effect was mediated by Fe itself and not by the transfection agent [202], a study carried out by Arbab et al. [203] proposed opposite conclusions. Subsequent studies confirmed that the internalization of SPIOs (ferumoxides) by hMSCs using a liposome transfection agent did not affect their chondrogenic, adipogenic, or osteogenic differentiation [204]. Lastly, Au et al. [205] detected that labeling with SPIOs did not affect the calcium-handling of cardiomyocytes derived from ESCs, suggesting the feasibility of* in vivo* tracking of ESCs inside the heart with these NPs detected via MRI, without affecting the cardiac differentiation potential and functional properties of ESCs.

SPIOs were used to label rabbit MSCs in order to determine their fate* in vivo* [200], as described in the next section, but before that, authors analyzed the NP-associated biosafety. After 12 hours of incubation with 25 *μ*g/mL SPIO NPs (ferumoxides) and protamine sulphate as transfection agent, more than 90% of cells were labeled and MTT cytotoxicity and proliferation assay demonstrated no significant decrease of proliferation of SPIO-labeled cells compared with unlabeled cells after 9 days. Moreover,* in vitro* differentiation analysis indicated that cell differentiation in adipogenic, osteogenic, and chondrogenic cell lineages was similar between labeled and unlabeled rabbit MSCs [200].

Further research conducted by Delcroix et al. [198] in 2009 used SPIO NPs coated with 1-hydroxyethylidene-1.1-bisphosphonic acid (HEDP) to label rat MSCs (rMSCs). Cells were incubated with NPs for 48 hours and authors established that more than 90% of cells contained enough iron to allow their detection without significant alteration of cell viability. Moreover, cellular ultrastructure was conserved and the differentiation potential toward osteogenic and neuronal lineages did not exhibit significant differences with unlabeled rMSCs. In the same work, authors evaluated the* in vivo* application of labeled cells, which will be discussed in next part of this review.

Since the use of transfection agents to efficiently label SCs is still an open issue for the biosafety of SPIOs, Ramos-Gómez et al. [206] have recently reported the development, optimization, and validation of an efficient procedure to label human NSCs with different commercial NPs in the absence of transfection agents. Authors evaluated the influence of different concentration of SPIOs on immortalized human NSCs after 72 hours of exposure and found that the dose of 50 *μ*g/mL did not affect cell survival, evaluated by MTT assay, and resulted in 80 to 90% labeling efficiency. They also analyzed whether the presence of SPIOs was compatible with a normal cell-cycle progression, showing that the distribution of the percentages of cells in G0-G1, S, and G2-M phases was the same in labeled and unlabeled cells. Finally, the stemness and differentiation potentials of hNSCs were not affected.

It is important to underline that different doses of SPIOs with different types of iron oxide cores and different coating layers have been used to label diverse cell types, thus not allowing for obtaining clear indications about the most useful conditions for their usage. For a better understanding of their* in vitro* biosafety, a standard procedure for SC labeling needs to be identified.

### 5.5. Gold NPs

The strongly enhanced surface plasmon resonance of Au NPs at optical frequencies makes them excellent scatterers and absorbers of visible light; moreover, Au NPs are characterized by ease of synthesis and conjugation to a variety of biomolecular ligands, antibodies, and other targets, making them suitable for use in biochemical sensing and detection, medical diagnostics, therapeutic applications, and SC tracking. In particular, the most used are gold nanorods [207]. One other important feature of gold NPs is their ability to be used for photothermal therapy (PTT), as they can be induced by infrared light, creating vibrational energy and heat which in turn kill unwanted cells [208].

For SC labeling, Ricles et al. [209] investigated the function of MSCs labeled with various formulations of Au NPs obtained by varying size and poly-L-lysin coating. Results demonstrated that loading of MSCs with Au nanotracers (NTs) did not alter cell function and, based on the inductively coupled plasma mass spectrometry results, long-term imaging and tracking of MSCs were feasible. The same group in 2012 [210] used noncoated 20 nm Au NTs to label MSCs. In order to analyze the sensitivity as well as quantification of the ultrasound and photoacoustic imaging method, labeled cells were seeded in a tissue mimicking gelatin phantom, a PEGylated fibrin gel, and they demonstrated that tracking of viable cells was possible over one week. The* in vivo* application will be discussed in the next section.

Jokerst et al. [211] used silica-coated Au nanorods (NRs) to label MSCs, demonstrating that silica facilitated NP uptake more than 5-fold and increased the photoacoustic signal of these NPs. Moreover, no toxicity or altered proliferation was observed and pluripotency in osteogenic and adipogenic lineage was retained. The analysis of the secretome derived form labeled hMSCs indicated that only interleukin-6 (IL-6) was dysregulated more than 2-fold from a pool of 26 cytokines.

More recently, Nam et al. [212] demonstrated that silica and poly-L-lysin coated SiO_2_ Au NRs were able to label adipose-derived SCs (ASCs) without affecting their viability.

A limitation of Au NPs is that they cannot be employed with imaging methods of higher resolution, such as MRI.

## 6. *In Vivo* Applications of Stem Cells Labeled with Nanoparticles

In general, NP technology applied for* in vivo* noninvasive SC tracking must allow long-term and sensitive localization of the cells avoiding cytotoxicity as much as possible.* In vivo* preclinical studies are mainly conducted on rodent models and are performed to optimize the use of NPs starting from the dose range validated in* in vitro* experiments.

In preclinical models, it is possible to compare in the same animal different injection sites and even to transplant both labeled and unlabeled cells in order to compare their behavior. Due to the noninvasive real-time analysis of labeled SCs inside animal models, it is conceivable not only to follow the migration of injected SCs but also to obtain information about the long-term outcomes of the treatment, that is, several months after cell injection without the sacrifice needed, having also the possibility to confirm the results obtained using other techniques like histological examination. Thanks to these characteristics, the use of NPs in* in vivo* preclinical experiments could give fundamental information about optimized dosages, preferred sites of engraftment, and specialized timing of SC injection, allowing for the tailoring of treatments to individual patients.

In particular, for regenerative medicine purposes, the most used modalities to track SCs* in vivo* are fluorescence, magnetic resonance, and photoacoustic imaging. We summarize in Table 2 the main studies concerning these different tracking methods.

### 6.1. Fluorescence Imaging

Several* in vitro* and* in vivo *studies have used NPs that can be detected using microscopic optical imaging techniques, including light and fluorescence, confocal and two-photon microscopy. Compared to other modalities, optical imaging presents several advantages such as accessibility for the majority of researchers, low cost, and high spatial and temporal sensitivity. See reference [213] for an overview on the recent development of optically active NPs for SC tracking. For these reasons, in preclinical studies, fluorescent imaging is currently the most used modality to follow SCs* in vivo*. In this concern, the most used fluorescent NPs to label cells for* in vivo* application are QDs, silica, and polymeric NPs.

Many authors have reported tracking of QD-labeled SCs* in vivo*; for example, Slotkin et al. [214] developed two techniques to directly label NSCs and progenitor cells (NSPCs) of the developing mammalian central nervous system with QDs* in vivo*. Using* in utero* ultrasound-guided injection or electroporation, they successfully QD-labeled ventricular zone (VZ) and subventricular zone (SVZ) NSPCs of the mouse embryonic telencephalon. After QD labeling, NSPCs appeared to continue normally developing, migrating, and differentiating, as assayed* in vivo* until embryonic day (E) 18.5 and in neurosphere assays* in vitro*. Furthermore, authors revealed that labeling of early mouse embryos with QDs could be used to mark developing cell populations over time.

In 2007, Rosen et al. [215] reported another approach to track hMSCs using intracellular QDs upon cardiac rat heart injection. They demonstrated that single QD-hMSCs could be easily identified in histologic sections to determine their location for at least 8 weeks following delivery* in vivo* and their 3D spatial distribution could be reconstructed.

More recently, a method for conjugation of high quantum efficiency, photostable, and multispectral QDs was developed for long-term tracking of endothelial progenitor cells (EPCs) with improved signal-to-noise ratios [216]. Specifically, authors conjugated fluorescent QDs to acetylated low-density lipoprotein (acLDL) and used these QD-acLDLs to label a specific CD34^+^ subpopulation of EPCs isolated from rat bone marrow and track them in a rat model of laser-induced choroidal neovascularization, thereby demonstrating their potential for tracking EPCs in ocular angiogenesis, a critical pathologic feature of several blinding conditions.

However,* in vivo* visualization of the classically used fluorescent dyes sometimes requires surgical or invasive procedures that limit in-man studies, essentially because deep tissue penetration is not obtainable with this optical approach. Recently, it has been reported that fluorescence imaging in the second near-infrared window (NIR-II, 1.0–1.4 *μ*m) might be an ideal strategy for* in vivo* imaging due to its deeper tissue penetration and higher temporal and spatial resolution in comparison with fluorescence imaging (both in the visible and the first near-infrared windows, NIR-I, 650–950 nm) and tomographic imaging such as MRI and positron emission tomography (PET) [138, 217]. For these reasons, after the biosafety evaluation of Ag_2_S QDs, Chen et al. [138] used the NIR-II imaging to dynamically visualize the migration and distribution of labeled hMSCs in response to stromal derived factor-1*α* (SDF-1*α*), an essential chemokine regulating SCs migration and homing [218], on cutaneous wound sites and studied their healing effect. To achieve this goal, they used a mouse wound healing model implanted with either unloaded or SDF-1*α*-loaded collagen scaffolds, respectively, followed by a comprehensive investigation on how the concentration and distribution of hMSCs on the wound site would affect the healing process. First, authors demonstrated that there were no differences in the tropism effect of SDF-1*α* on the migration of labeled and unlabeled cells. Then, after* in vivo* application, they observed a higher intensity of fluorescence into the wound when the SDF-1*α*-loaded collagen scaffold was implanted, indicating that hMSCs were more actively recruited to the wound site and they were homogenously distributed. Further, this homogenous distribution of hMSCs in the wound area promoted the healing process, as shown by optimal wound closure and thickness of the regenerated epidermis. The authors [138] underlined that the novel Ag_2_S QDs NIR-II fluorescence imaging method they developed might turn out to be useful for future* in vivo *studies.

### 6.2. Magnetic Resonance Imaging

MRI is a largely noninvasive method for human* in vivo* imaging that uses a powerful magnetic field to align the nuclear magnetization of hydrogen atoms which are responsible for the majority of MR signals [219, 220]; hence, water distribution inside the body is shown by MRI and correlates with the anatomy of the body [221]. MRI is often used for diagnosis of different diseases and to identify cancer metastasis and inflammation sites [222]. Optimizing scanner parameters or using MRI contrast agents can enhance contrast between tissues of interest, allowing for clearer imaging of specific molecules, cells, or tissues. Indeed, although MRI has become one of the main imaging techniques used in oncology, its resolution is mostly insufficient at a molecular and cellular scale, unless magnetic contrast agents are employed [223].

Magnetic NPs could be categorized as T1 or T2 contrast agents for MRI depending on the relaxation processes. T2 contrast agents include SPIO NPs, bimetallic ferrite NPs (e.g., CoFe_2_O_4_, MnFe_2_O_4_, and NiFe_2_O_4_), and hybrid magnetic NPs such as Fe_3_O_4_-Au dumbbell NPs [224]. T1 contrast agents are primarily gadolinium- (Gd-) containing NPs (e.g., Gd-chelated lipid NPs, Gd-chelated dextran NPs) and gadolinium oxide NPs [224].

Magnetic NPs are generally used to label SCs before injection in essentially two ways: (a) surface labeling, in which NPs are attached to the cell surface, and his method is very useful for* in vitro* cell separation but in general it is unsuitable for* in vivo* purposes due to rapid reticuloendothelial recognition and subsequent elimination of labeled cells [225]; (b) internalization of NPs via endocytosis or phagocytosis.

In general, these contrast agents are applied for SC tracking because they give a strong signal, offer a direct and clear cell labeling, and allow noninvasive* in vivo* scanning. The details concerning these issues can be found elsewhere [213, 226].

Different from T2 agents that produce negative signal (dark spots) on MRI images, Gd-based T1 agents restitute bright positive signal. As they are bigger compared with conventional Gd chelates, gadolinium oxide NPs should warrant higher cell uptake and longer intracellular retention [227]. However, gadolinium oxide NPs are currently poorly used for cell tracking due to still insufficient understanding of their stability in cells and their possible effects on cell function [181] and, prior to applying Gd NPs in SC therapy, their effects on SC function need to be fully investigated. As a result, to date, Gado*CellTrack* is the only commercially available Gd-based NP used for experimental* in vivo* cell tracking.

SPIOs have been used as MRI contrast agents since 1990 [228]. Despite being not initially developed for cell labeling and tracking, they have been successfully adapted for tracking SCs after their transplantation [195, 229].

Delcroix et al. [198] investigated how SC tracking in neural migratory pathways could be potentially used for clinical therapy. It was already established that MSCs are maintained in an appropriate environment, composed of specific matrix molecules and growth factors, and able to transdifferentiate into neural cells* in vitro*, though very few works were focused on the* in vivo* counterpart. To overcome this limitation, the authors assessed the migratory potential of labeled rMSCs* in vivo* in response to brain neurogenic stimuli. In particular, cells labeled with HEDP-coated SPIOs were transplanted into the SVZ of either damaged or nondamaged rat brains and their migratory activity was assessed. A long-range migration distance of labeled rMSCs was observed in damaged brains, while no migration was observed in normal brains. Taken together, this research demonstrated for the first time that neural migratory pathways could be established and mapped out using SPIO NPs; however, a limitation of this study was that the migratory map could only be obtained through Prussian Blue staining to show iron presence on histological sections, whereas the* in vivo* detection of migrating rMSCs via MRI was precluded by the fact that the mechanical lesion induced a very important background hyposignal. According to the authors, precautions will have to be taken in studies seeking to visualize SPIOs-labeled cells although they did not observe any adverse effect in the behavior of the animals and no signs of toxicity were detected in histological sections 21 days after transplantation, therefore confirming the safety of SPIO NPs for* in vivo* applications.

Success in SPIO detection with MRI has also been reported in a rat model of Huntington's disease [196]. Authors investigated the effects of SPIO NP (ferumoxides) on human central nervous system SCs grown as neurospheres (hCNS-SCns). First, they showed that SPIOs-labeled hCNS-SCns proliferated and differentiated normally* in vitro* and exhibited neuronal electrophysiological characteristics. Furthermore, SPIO-labeled hCNS-SCns were then transplanted into newborn and adult (injured and uninjured) rodent brains and with both MRI and histology the authors found that transplanted cells exhibited a similar extent of survival, migration, integration, and differentiation with respect to their unlabeled counterparts.

Comparable results were seen in a study by Hu et al. [230], in which human umbilical cord MSCs (hUC-MSCs) labeled with SPIOs were transplanted surgically into the spinal cord of adult rats one day after spinal cord injury.* In vivo* MRI conducted 1 and 3 weeks after cell injection showed a large reduction in signal intensity in the region transplanted with SPIOs-labeled hUC-MSCs with respect to unlabeled cells. Transplanted hUC-MSCs engrafted within the injured spinal cord and survived for at least 8 weeks, led to reduced spinal cord injury, and promoted muscular-skeletal recovery if compared with the control group in which no cells were injected.

Jing et al. [200] after the biosafety study of SPIOs (ferumoxides) on rabbit MSCs already reported further tracked labeled cells by* in vivo* MRI. Labeled autologous rabbit MSCs were seeded in chitosan and glycerophosphate (C-GP) gels and then injected into the knee joint cavity of rabbit articular cartilage defect models. Results showed marked hypointense signal void areas representing injected MSCs that could be observed up to 12 weeks. Labeled cells migrated into the synovial fluid at the suprapatellar bursa, the popliteal space site, and subchondral bone of femur; however, no cells were detected in the defect after 12 weeks, suggesting that autologous MSCs did not actively participate in the repair of articular cartilage defects following intra-articular injection.

Amsalem and collaborators [231], studying the role of SCs in myocardial repair, sought to determine whether the outcome of injected MSCs could be affected by SPIO NP (ferumoxides) labeling in a rat model of myocardial infarction. The authors found that after 1, 2, and 4 weeks, MRI signal showed well-defined hypointense areas in animals treated with SPIO-labeled MSCs, while control specimens injected with either saline or unlabeled MSCs showed no areas of hypointensities. Retention of the magnetic signal throughout the 4-week period was observed in all the treated rats irrespective of myocardial infarction induction.

Chapon et al. [232] used dextran-coated SPIO NPs to track bone marrow MSCs and determined their effect in host cardiac tissue after myocardial infarction. They showed that the SPIO-labeled MSCs could be easily tracked using MRI within the first week following myocardial infarction and cell implantation, while after a few weeks the signal voids were variable. This may be explained by the dispersion/migration of the labeled cells after implantation. In addition, radiolabeled glucose (2-deoxy-2-[F-18] fluoro-D-glucose [FDG]) and positron emission tomography (PET) were used to determine glucose uptake of the infarcted areas, showing a greater amount in the hearts transplanted with bone marrow MSCs compared to infarcted hearts that did not receive the cells. However, the early increase in FDG uptake did not persist 6 weeks after infarction, probably as a result of death of transplanted cells; finally, no improved left ventricular function was observed in treated rats. Altogether, this study highlighted both the ability to track SCs by noninvasive imaging and the importance of using multimodal platforms to establish the effect of SCs on cardiac function. It is not clear at the moment what the mechanism responsible for the (limited) clinical efficacy observed following injection of SCs into the damaged heart is or whether this might be due to either a cellular or a humoral response.

In another study of Blocki et al. [233], to overcome the limited efficacy of cardiac SCs-based therapy due to poor cell retention, injectable microcapsules were developed for the delivery of MSCs into the infarcted area. For* in vivo* applications, rat MSCs were first labeled with the same dextran-coated SPIO NPs used by Chapon et al. [232]. Labeled cells were then injected either as a single cell suspension or within microcapsules into the injured tissue, as well as in the peri-infarct area. MSCs injected as single cells were either not detected at all or only at an early time point after surgery (two days). In contrast, encapsulated labeled MSCs could be tracked for the whole duration of the study (six weeks), although signal intensity decreased over time. Moreover, a fraction of transplanted labeled MSCs were able to migrate out of the microcapsules and integrate into the host tissue.

As already discussed in Section 5, Ramos-Gómez et al. [206] optimized the labeling of hNSCs with different SPIO NPs without transfection agents; labeled cells were then transplanted into the right striatum of a rat model of Parkinson's disease, while unlabeled cells were delivered into the left striatum as an internal imaging control. MRI was performed at different time points after cell transplantation (48 h, 2, 4, and 8 weeks), the intensity and size of the signal decreased slightly during the time period studied, and SPIOs NP labeled cells were still clearly detectable after 8 weeks. Moreover, 5 months after cell delivery, a clear hypointense signal was still visible at the site of transplantation. However, according to the authors, long-term MR images should be interpreted with caution due to the possibility that some SPIO NPs may have been expelled from the transplanted cells and internalized by host microglial cells.

An example of application of silica shell on magnetic NPs (MNP) containing rhodamine B isothiocyanate was made by Kim et al. [234]. Authors succeeded in tracking intrasplenically injected bone marrow MSCs labeled with fluorescent MNP in a liver cirrhosis rat model. As reported in a previous section, Zhang et al. [162] fabricated fluorescent mesoporous silica-coated superparamagnetic iron oxide NPs (fmSiO_4_@SPIONs) for neural stem cell (C17.2) MRI. These NPs were discrete and uniform in size and had a clear core-shell structure. When implanted into the right hemisphere of mice subjected to stroke, contralateral to the ischemic territory, a small amount of labeled cells could be tracked while migrating to the injury sites using a clinical MRI scanner (3 T). Remarkably, labeled cells could be monitored while homing to the ischemic area even when administered intravenously. MRI observations were finally corroborated by fluorescence-based histological sections from brain tissue [162].

A limitation to the use of SPIO NPs is their occasional extracellular deposition in tissues, either by active exocytosis or by passive release due to death of transplanted cells. Indeed, Berman et al. [235] transplanted SPIO NP-(BioPAL) labeled C17.2 NSCs into immunodeficient (graft-accepting* Rag2*) or immunocompetent (graft-rejecting Balb/c) mice and observed hypointense voxel signals and bioluminescence emission over a period of 93 days. Unexpectedly, in mice that rejected cells, the hypointense MR signal persisted throughout the entire time course, whereas in the nonrejecting mice the contrast cleared at a faster rate. In immunocompetent mice, infiltrating leukocytes and microglia were found to surround dead cells and to internalize superparamagnetic iron oxide clusters thus ensuring contrast retention, whereas in immunodeficient mice proliferation of surviving transplanted cells and associated label dilution was supposed to be at the base of contrast clearance. Thus, interpretation of signal changes during long-term MR cell tracking is complex and requires caution. Furthermore, Terrovitis et al. [236] studied nonimmunoprivileged human and rat cardiac-derived SCs and hMSCs labeled with both SPIO NPs (ferumoxides) and *β*-galactosidase and injected intramyocardially into immunocompetent Wistar-Kyoto rats. On day 2, injection sites of xenogeneic and syngeneic cells (cardiac-derived SCs and MSCs) were identified by MRI as large intramyocardial signal voids that persisted for 3 weeks. Histological sections revealed after 3 weeks the presence of iron-containing macrophages at the injection site (identified via specific CD68 staining), but very few or no *β*-galactosidase-positive SCs in animals transplanted with syngeneic or xenogeneic cells, respectively. These results indicated that MRI of ferumoxide-labeled cells did not reliably report on long-term SCs engraftment in the heart, as deposited iron particles were scavenged by macrophages, which could then generate a false signal on MRI.

As an example of their clinical use, SPIO NPs (ferumoxides) were used by Janowski et al. [237] for MRI-tracking of autologous cord blood nucleated cells in a nine-month-old patient in a permanent vegetative state as a result of global cerebral ischemia. Cell transplantation into the right frontal horn of the lateral ventricle resulted in a focal deposition of cells within the ipsilateral occipital horn, as detected in 24 h after operative MR scans. Over a period of 4 months, cell location within the occipital horn did not change, although a gradual decrease of signal was apparent. Very few hypointense regions were observed in other parts of the ventricular system, including the contralateral ventricle or the fourth ventricle. At the long-term follow-up visit (33 months after transplantation), SPIO-related hypointense signals were no longer detectable; moreover, MRI at that time did not detect any abnormality that could be attributed to tumor or overgrowth of transplanted cells and finally no major changes in the patient's neurological status were observed. Despite the poor prognosis, the patient survived without deterioration for an additional three years. This study is one of the first clinical MRI cell tracking trials that aimed to demonstrate the feasibility of this approach in man. Authors underlined that an external magnet may possibly be used to direct SPIO-labeled cells within a fluid compartment such as the ventricular system.

Another important limitation of SPIO NP application is that their generated contrast can easily be confounded with other contrast sources such as bleedings or blood vessels [238]. Furthermore, since contrast is achieved indirectly through disturbances of the local magnetic field experienced by surrounding hydrogen nuclei, quantification of the number of cells* in vivo *is questionable [239]. To overcome these limitations, fluorine (^19^F) MRI technique could be used, but at the cost of lower sensitivity [240]. The interest in ^19^F MRI has been recently increased by the availability of an FDA-approved ^19^F nanoemulsion [241]. Perfluorocarbons (PFCs) have many fluorine atoms with identical chemical shifts and for this reason they are the most commonly used for ^19^F MRI cell tracking applications; PFCs include perfluoro-15-crown-5-ether, linear perfluoropolyethers, and perfluorooctyl bromide [242, 243].

Boehm-Sturm et al. [244] labeled human NSCs with perfluoropolyether and adult male CD1 mice that were implanted into the striatum with an injection of either labeled or unlabeled cells. Authors detected significant ^19^F signal from the grafts with labeled cells in all animals, while no signal was detected from implanted nonlabeled control cells. In mice with labeled cells grafted in both hemispheres, the ^19^F signal persisted at least 6 days after implantation and quantitative signal-to-noise ratio (SNR) analysis revealed that total ^19^F SNR decreased by <20% from day 2 to day 6. A detection limit of 10,000 cells was found* in vivo* and the location and density of human cells (hunu^+^) on histological sections correlated well with observations in the ^19^F MR images. Furthermore, no label-related changes in the numbers of Ki67, nestin, GFAP, or bIII-tubulin^+^ cells were detected on histological sections. However, thorough long-term evaluations of both effects of PFCs on grafted SCs and efficacy of the labeled cells in the pathological brain, for example, stroke models, will be needed in the future.

Overall, there is considerable promising and established research that demonstrates that magnetic NPs have an exciting role in SC tracking by noninvasive mechanisms which could, in time, lead to deeper appreciation of tissue regeneration pathways and future clinical applications.

### 6.3. Photoacoustic Imaging

Photoacoustic imaging provides high detection sensitivity, which allows imaging of down to 100,000 cells* in vivo* and high spatial and temporal resolution which are at least an order of magnitude below traditional cell imaging techniques, such as PET [211]. In photoacoustic imaging, a photoacoustic wave is generated by thermal expansion of tissue after absorption of a short laser pulse. The magnitude of the photoacoustic wave is proportional to the laser fluency and to the optical absorption coefficient [245].

Because of its low cost, deep penetration (up to 2 cm), noninvasiveness, and good resolution (100 *μ*m), photoacoustic imaging is becoming an alternative method to fluorescent, MRI, and radioactive imaging for SC* in vivo* tracking [209–211].

Au NPs and Au nanorods are potential contrast agents for photoacoustic imaging [246, 247].

Jokerst et al. [211] used silica-coated Au NRs to label MSCs before intramuscular injection in mice and obtained a cell detection limit* in vivo* of 100,000 cells, which was well below the clinically relevant numbers.

Nam et al. [210] used a PEGylated fibrin gel containing Au NP-labeled MSCs to inject intramuscularly the lateral gastrocnemius of anesthetized Lewis rats and the contrast brought by Au NPs allowed the researchers to visualize the* in vivo* localization of labeled MSCs using photoacoustic imaging. The authors also performed a longitudinal* in vivo* monitoring of the spatial distribution of labeled MSCs at days 3, 7, and 10 after injection, demonstrating the feasibility of this approach. The same group in 2014 [248] produced a dual Au NP system which was capable of monitoring both delivered MSCs and infiltrating macrophages using photoacoustic imaging.* In vitro* analysis confirmed preferential labeling of the two cell types with their respective Au NPs and the maintenance of cell function following labeling with NPs. In addition, delivery of the system within a rat hind limb ischemia model demonstrated the ability to monitor SCs and distinguish and quantify macrophage infiltration. These findings were confirmed by histology and mass spectrometry analysis.

Furthermore, Nam et al. [212] introduced a novel application of combined ultrasound and photoacoustic imaging to assess both burn injury and skin tissue regeneration. Authors used silica and poly-L-lysin coated SiO_2_ NRs, previously discussed in Section 5, to label ASCs engrafted within PEGylated fibrin gel and then implanted in a rat model of cutaneous burn injury. The labeled ASCs were successfully tracked up to 2 weeks and were distinguished from host tissue components (e.g., epidermis, fat, and blood vessels) through spectroscopic photoacoustic imaging. Imaging-based analysis demonstrated ASCs localization in the top layer of the skin and a higher density of regenerating blood vessels in the treated groups.

Overall, these data show that ultrasound and photoacoustic imaging-based strategies coupled with Au NRs have a great potential for SC therapy and tissue engineering due to noninvasiveness, safety, selectivity, and ability to provide long-term monitoring.

## 7. Conclusions

Overall, the results collected in this review show that several NP-based imaging techniques may potentially highlight the position of transplanted SCs and further that, by combining different imaging techniques such as computed tomography, PET, single-photon emission computed tomography (SPECT), and optical imaging, or ultrasound and photoacoustic imaging, a wider array of data could be gathered to present a clearer picture of biodistribution, differentiation, cell viability, and function of the cells inside the host.

Indeed, when choosing appropriate SC tracking agents, it is important to consider their respective imaging requirements. The use of magnetic NPs often requires complex imaging systems, such as MRI, whereas use of fluorescent NPs relies on optical imaging, which may be more accessible to the majority of researchers. On the other hand, poor tissue penetration of fluorescence imaging might mostly limit this approach to preclinical analyses. Interestingly, over the last years, photoacoustic imaging seems to offer low cost, deep penetration, and good resolution noninvasive imaging characteristics and it is becoming an alternative method for SC labeling and* in vivo *tracking.

Due to contrasting results from cytotoxicity analysis of various NPs, prior to their therapeutic use as contrast agents for SC tracking, it is becoming increasingly important to conduct systematic* in vitro* and* in vivo* studies to assess the toxicological profiles of the chosen NPs and to evaluate their potential influence on the self-renewal and differentiation properties of SCs. Future research aimed at optimization of methods to determine NP toxicity on SCs will be fundamental to obtain reproducible data and allow stable conclusions in this emerging field.

In particular, the development of both* in vitro* and* in vivo* nanotoxicology methods should consider specific aspects, among them, (a) detailed characterization of the specific NP in terms of composition, shape, size, surface charge, and content of the outside protein* corona*; (b) standardization of both toxicity assays and methods to produce the samples; and (c) survey of interferences and discrepancies between NPs and specific assays and/or their detection methods.

## Figures and Tables

**Table 1:** Summary of different *in vitro* biosafety studies performed using nanoparticles.

Study	Stem cell	Species	Nanoparticle	Labeling dose	Analysis	Main results of analysis
Chakraborty et al. 2007 [131]	MSC	Human	QDs	250 pm–16 nM	Differentiation potential	No adverse effects

Shah et al. 2007 [132]	MSC	Human	QDs	20–50 nM	Cell viability, differentiation potential	No adverse effects

Hsieh et al. 2006 [133]	MSC	Human	QDs	1.625 *μ*g	Cell viability, differentiation potential	Chondrogenic differentiation impairment

Hsieh et al. 2006 [134]	MSC	Human	QDs	1.625 *μ*g	Cell cycle distribution, differentiation potential	Osteogenic differentiation impairment

Wang et al. 2015 [136]	MSC	Human	QDs	0.75–3 *μ*g/mL	Cell viability, immunophenotypic profiles	No adverse effects

Shah and Mao 2011 [137]	MSC	Human	QDs	Range of doses	Selective labeling	No adverse effects

Chen et al. 2015 [138]	MSC	Human	QDs	12.5 *μ*g/mL	Cell viability, proliferation, stemness, and differentiation potential	No adverse effects

Huang et al. 2005 [157]	MSC	Human	Silica	20 *μ*g/mL	Cell viability, proliferation, immunophenotypic profiles, differentiation potential, and discrimination live/apoptotic cells	No adverse effects

Hunag et al. 2008 [159]	MSC	Human	Silica	20 *μ*g/mL	Uptake efficiency and mechanism	Serum influences cell uptake efficiency

Accomasso et al. 2012 [154]	MSC	Human	Mesoporous silica	Unknown	Cell viability, proliferation, immunophenotypic profiles, differentiation potential, and uptake mechanism	No adverse effects

Lesniak et al. 2012 [155]	MSC	Human	Mesoporous silica	100 *μ*g/mL	Cell viability, differentiation potential	No adverse effects

Catalano et al. 2015 [156]	MSC	Human	Mesoporous silica	40 *μ*g/mL	Cell viability, actin organization, and differentiation potential	No adverse effects, but enhancement of actin polymerization

Liu et al. 2008 [161]	MSC	Human	Mesoporous silica core-shell SPIO	3–10 *μ*g/mL	Uptake efficiency, cell viability, and differentiation potential	No adverse effects

Zhang et al. 2013 [162]	Neuronal progenitor line	Mouse	Mesoporous silica core-shell SPIO	5–33 *μ*g/mL	Uptake efficiency, cell viability, and differentiation potential	No adverse effects

Jiang et al. 2010 [172]	MSC	Human	Polystyrene	7.5 *μ*g/mL	Uptake efficiency and mechanism	Amino groups on NP surface influence uptake mechanism

Huang et al. 2015 [178]	MSC	Porcine	Polystyrene	0.2 mg/mL	Uptake efficiency, cell viability	Little cytotoxicity

Bulte et al. 2004 [202]	MSC	Human	SPIO (ferumoxides)	13–16 pg Fe/cell	Cell viability, differentiation potential	Chondrogenic differentiation impairment

Arbab et al. 2005 [203]	MSC	Human	SPIO (ferumoxides)	25 *μ*g/mL	Lysosomal degradation	Chondrogenic differentiation impairment due to transfection agent, not SPIO

Song and Ku 2007 [204]	MSC	Human	SPIO (ferumoxides)	25 *μ*g/mL	Cell viability, differentiation potential	No adverse effects

Au et al. 2009 [205]	ESC	Mouse	SPIO	50 *μ*g/mL	Differentiation potential and calcium handling	No adverse effects

Jing et al. 2008 [200]	MSC	Rabbit	SPIO (ferumoxides)	25 *μ*g/mL	Cell viability, proliferation, and differentiation potential	No adverse effects

Delcroix et al. 2009 [198]	MSC	Rat	SPIO	25 *μ*g/mL	Cell viability, morphology, and differentiation potential	No adverse effects

Ramos-Gómez et al. 2015 [206]	Neuronal line	Human	SPIO (different types)	50 *μ*g/mL	Cell viability, cell cycle distribution, and differentiation potential	No adverse effects

Ricles et al. 2011 [209]	MSC	Human	Gold	10^12^ NPs/mL	Uptake efficiency	No adverse effects

Nam et al. 2012 [210]	MSC	Human	Gold	10^12^ NPs/mL	Uptake efficiency, *in vitro* cell tracking	No adverse effects

Jokerst et al. 2012 [211]	MSC	Human	Silica-coated gold	0.0–0.14 nM	Cell viability, proliferation, differentiation potential, and secretome	No adverse effects, but IL-6 dysregulation

Nam et al. 2015 [212]	Adipose derived	Rat and human	Silica-coated gold	4*∗*10^7^ NPs/cell	Cell viability	No adverse effects

**Table 2:** Summary of different *in vivo* studies performed with nanoparticles used to label stem cells.

Study	Experimental model		Nanoparticle		Stem cell			Labeling approach	Main results
	Animal	Type	Type	Dose	Source	Type	Number		
Slotkin et al. 2007 [214]	Mouse	Developing central nervous system	QDs	2.5 ng	Mouse	Neural stem and progenitor cells	Direct labeling	Fluorescence imaging	Novel *in utero* electroporation and ultrasound-guided *in vivo* delivery, QDs are compatible with early mammalian embryonic development

Rosen et al. 2007 [215]	Rat	Normal	QDs	8.2 nM	Human	MSC	1 *∗* 10^5^	Fluorescence imaging	Long-term tracking technique for at least 8 weeks permits the complete three-dimensional reconstruction of the locations into the heart

Chen et al. 2015 [138]	Mouse	Wound healing model	QDs	12.5 *μ*g/mL	Human	MSC	1 *∗* 10^5^	Fluorescence imaging	Dynamic visualization *in vivo* of migration to the wound after intravenous injection, treatment of wound with collagen scaffold-SDF-1*α* enhances the reepithelialization and the neovascularization and accelerates the wound healing

Delcroix et al. 2009 [198]	Rat	Lesioned brain	SPIO	25 *μ*g/mL	Rat	MSC	40–100 *∗* 10^3^	Magnetic resonance imaging	Cell migration toward lesions and description of the long distance migration from the SVZ toward the olfactory bulb through the rostral migratory stream

Guzman et al. 2007 [196]	Rat	Huntington's disease model	SPIO (Ferumoxides)	5 *μ*g/mL	Human	Central nervous system cell	1 *∗* 10^5^	Magnetic resonance imaging	Long-term survival and differentiation of cells

Hu et al. 2012 [230]	Rat	Spinal cord injury	SPIO	7 *μ*g Fe/mL	Human	MSC	4 *∗* 10^5^	Magnetic resonance imaging	hUC-MSCs can survive and migrate in the host spinal cord after transplantation, which promote functional recovery after spinal cord injury

Jing et al. 2008 [200]	Rabbit	Articular cartilage defect model	SPIO (ferumoxides)	25 *μ*g/mL	Rabbit	MSC	1 *∗* 10^8^	Magnetic resonance imaging	Engineered autologous MSCs do not actively participate in the repair of articular cartilage defects following intra-articular injection

Amsalem et al. 2007 [231]	Rat	Myocardial infarction	SPIO (ferumoxides)	25 *μ*g/mL	Rat	MSC	2 *∗* 10^6^	Magnetic resonance imaging	MSCs attenuated progressive left ventricular dilatation and dysfunction compared with controls without cells

Chapon et al. 2009 [232]	Rat	Myocardial infarction	SPIO	10 *μ*g/mL	Rat	MSC	5 *∗* 10^5^	Magnetic resonance imaging	Ability to track SCs by noninvasive imaging, and the importance of using multimodal platforms to establish the effect of SCs on cardiac function

Blocki et al. 2015 [233]	Rat	Myocardial infarction	SPIO	10 *μ*g/mL	Rat	MSC	1 *∗* 10^6^	Magnetic resonance imaging	Injectable microcapsules for the delivery overcome current limitations of poor cell retention in cardiac cell-based therapy

Ramos-Gómez et al. 2015 [206]	Rat	Parkinson's disease model	SPIO (different types)	50 *μ*g/mL	Human	Neuronal line	3 *∗* 10^5^	Magnetic resonance imaging	Feasibility for long-term tracking, possible internalization of NPs by host microglial cells

Kim et al. 2010 [234]	Rat	Liver cirrhosis model	Silica shell on SPIO (ferumoxides)	100 *μ*g/mL	Human	MSC	3 *∗* 10^6^	Magnetic resonance and fluorescence imaging	Feasibility for tracking in liver cirrhosis model

Zhang et al. 2013 [162]	Mouse	Stroke model	Mesoporous silica core-shell SPIO	5–33 *μ*g/mL	Mouse	Neuronal progenitor line	1 *∗* 10^5^	Magnetic resonance and fluorescence imaging	Monitoring of the cell homing to the ischemic area after intravenously injection

Berman et al. 2011 [235]	Mouse	Immunodeficient and immunocompetent	SPIO (BioPAL)	25 *μ*g/mL	Mouse	Neuronal progenitor line	3 *∗* 10^5^	Magnetic resonance and bioluminescence imaging	Live cell proliferation and associated label dilution may dominate contrast clearance as compared with cell death and subsequent transfer and retention of superparamagnetic iron oxide within phagocytes and brain interstitium

Terrovitis et al. 2008 [236]	Rat	Myocardial transplantation model	SPIO (ferumoxides)	25 *μ*g/mL	Rat and human	Cardiac-derived stem cell	5 *∗* 10^5^ 7.5 *∗* 10^5^	Magnetic resonance imaging	Persistence of significant iron-dependent MRI signal derived from ferumoxide-containing macrophages despite few or no viable stem cells 3 weeks after transplantation

Janowski et al. 2014 [237]	Human	Permanent vegetative state	SPIO (ferumoxides)	100 *μ*g/mL	Human	Cord blood nucleated cell	3.6 *∗* 10^7^	Magnetic resonance imaging	Feasibility of long-term clinical tracking

Boehm-Sturm et al. 2011 [244]	Mouse	CD-1	Perfluoropolyether	120 mg/mL	Human	Neural stem cell	1.5 *∗* 10^5^	Magnetic resonance imaging	^19^F MRI can be utilized for tracking human NSCs in brain implantation studies

Jokerst et al. 2012 [211]	Mouse	Normal	Silica-coated gold	0.0–0.14 nM	Human	MSC	Unknown	Photoacoustic imaging	Feasibility of multimodal approach

Nam et al. 2012 [210]	Rat	Normal	Gold	10^12^ NPs/mL	Human	MSC	5 *∗* 10^4^	Photoacoustic imaging	Multimodal approach capable of noninvasive, sensitive, quantitative, and longitudinal assessment of stem cell behaviors with high spatial and temporal resolutions at sufficient depths

Ricles et al. 2014 [248]	Rat	Hind limb ischemia model	Gold	10^12^ NPs/mL	Rat	MSC	1 *∗* 10^4^	Photoacoustic imaging	System capable of monitoring both delivered stem cells and infiltrating macrophages using photoacoustic imaging

Nam et al. 2015 [212]	Rat	Cutaneous burn injury model	Silica-coated gold	4 *∗* 10^7^ NPs/ cell	Rat and human	Adipose derived	1 *∗* 10^6^	Photoacoustic imaging	Feasibility of long-term tracking, ability of multimodal imaging to assess both burn injury and skin tissue regeneration
